# Articulated coordination of the right arm underlies control of bow parameters and quick bow reversals in skilled cello bowing

**DOI:** 10.3389/fpsyg.2014.00885

**Published:** 2014-08-19

**Authors:** Julius Verrel, Marjorie Woollacott, Ulman Lindenberger

**Affiliations:** ^1^Center for Lifespan Psychology, Max Planck Institute for Human DevelopmentBerlin, Germany; ^2^Department of Human Physiology and Institute of Neurosciences, University of Oregon, Eugene, ORUSA

**Keywords:** coordination, degrees of freedom, freezing, expertise, cello bowing, music performance, movement reversal

## Abstract

Stringed instrument bowing is a complex coordinative motor skill acquired though years of intense practice. We apply a novel “freezing” analysis to investigate how movement at different joints contributes to bow transport (movement amplitude), stabilization of bow parameters (angle, velocity) during bow movements, and quick reversals of bow direction (acceleration amplitude). Participants were ten advanced or professional cellists (19–32 years, at least 10 years of practice) and ten age-matched novice players. Arm and bow movements were recorded using 3D motion capture. To assess how performance depends on articulated use of the right arm, actual data were compared to surrogate data, generated by artificially removing movement at (“freezing”) individual joints in measured arm movements. This analysis showed that both elbow and shoulder significantly contribute to bow transport in experts, while only the shoulder contributed to bow transport in novices. Moreover, experts showed more strongly increased variability of bow parameters and reduced acceleration amplitudes at bow reversals for surrogate compared to actual movement data. This indicates that movement across joints was organized to reduce bow variability and achieve quick bow reversals. Corresponding effects were less pronounced or absent in the novices, in particular for the wrist and elbow. Our results demonstrate the importance of articulated use of the right arm and clarify the contribution of different joints in experts’ bowing performance. Moreover, they support theories of motor control and learning that propose exploitation of biomechanical degrees of freedom, in particular of distal joints, as a critical component in skilled motor performance.

## INTRODUCTION

Theoretical and empirical work on motor control and learning indicates that successful motor performance depends on task-specific coordination of multiple degrees of freedom across the body. Moreover, it has been suggested that initial stages of acquiring a new skill are characterized by a “freezing” of degrees of freedom (i.e., eliminating or minimizing motion at particular joints) which are gradually released as learning progresses to achieve more flexible and efficient performance (Bernstein, 1967, 1996; Vereijken et al., 1992). Due to interaction torques occurring between body segments, freezing joints is likely an active and effortful mechanism, achieved by muscular co-contraction. However, by reducing the number of degrees of freedom which need to be coordinated in a task, freezing is hypothesized to simplify control (at the expense of efficiency) at early stages of skill acquisition. In the present study, we apply a novel “freezing” analysis to characterize the relevance of coordinated use of degrees of freedom for performance in a complex motor task, bow control in cello playing.

We have previously shown that expert cellists show better control of bow parameters (e.g., bowing angle and bow velocity) as well as enhanced use and coordination of distal degrees of freedom compared to novice cellists (Verrel et al., 2013a). Moreover, it has been shown that expert cellists show high-amplitude acceleration profiles along with temporally differentiated sequential coordination of the right arm at bow reversals (Verrel et al., 2013b). However, these analyses are mostly correlational and do not make a direct functional link between performance observed at the level of the bow movement and the underlying inter-joint coordination.

In the present study, we use a novel approach to investigate the relationship between coordination of degrees of freedom (joint angles) and the resulting movement of the end effector (the bow). The relationship is analyzed by comparing empirically observed data with surrogate data, in which movement at individual joints is artificially removed by replacing the data for that joint by a constant value. The effect of this “freezing” of the joint is assessed at the level of performance variables: if performance is degraded by eliminating motion at a joint, this indicates that this joint was originally moved and coordinated with the other joints in a way to achieve a certain performance criterion.

As a general approach, this method has been previously suggested to quantify contribution of different degrees of freedom to range of motion of an end effectors or whole-body center of mass (Park et al., 2005, 2008). In the present analysis, we extend the method to more complex measures of performance suggested by previous studies on skilled cello bowing (Verrel et al., 2013a,b): variability of bow velocity and bowing angle *during bow movements*, as well as acceleration amplitude *at bow reversals.* As low variability in bow velocity and bowing angle is indicative of good performance, use and coordination of a joint would be considered important for stabilizing that bow parameter if freezing that joint leads to an increase in variability. Similarly, as quick bow reversals require high acceleration amplitudes, motion at a joint would be considered important for achieving quick bow reversals if freezing that joint leads to a reduction in acceleration amplitude.

In the present study, we re-analyze cello bowing data from a previous study (Verrel et al., 2013b) by the approach described above. The effect of use and coordination of different joints of the right arm on cello bowing performance is assessed in expert and novice cellists, and compared between the two groups. In line with the research discussed above as well as cello technique literature (Mantel, 1995), we predicted that the effect of removing motion at (“freezing”) individual joints on bowing performance would be more pronounced in experts than in novices, in particular for more distal joints.

## MATERIALS AND METHODS

### PARTICIPANTS

Ten advanced or professional players (3 female, age ± SD: 22.9 ± 4.3 years, age range: 19–32 years) and 10 novice players (3 female, 23.5 ± 3.5 years, age range 21–32 years) took part in the study. Advanced or professional players (“experts”) had at least 5 years of cello education (12.4 ± 5.5 years, range: 5–20 years), at least 10 years of total cello playing experience (14.4 ± 5.1 years, range: 10–24 years), and were students of cello at a conservatory or advanced amateurs. Novices had no prior experience with the cello or any other bowed string instrument. The experiment was approved by local ethics committees (Max Planck Institute for Human Development, Berlin, and University of Oregon, Eugene) and conducted with participants’ written informed consent and in accordance with the Declaration of Helsinki.

### EXPERIMENTAL PROCEDURE

At the beginning of the experiment, novices received a standardized introduction to cello bowing, approved by a highly experienced cello teacher (Steven Pologe, School of Music and Dance, University of Oregon). This included instructions on how to hold the bow with the right hand, controlling the position and movement of the bow relative to the string, and controlling bowing velocity. In particular, novice participants were explicitly instructed to maintain an orthogonal angle and constant contact point between bow and string, and to move the bow at a constant velocity. For participants without any prior musical experience, additional instructions and practice were given regarding timing their own movements with the metronome. Special care was taken to prevent fatigue or injury in carrying out the unfamiliar movement, by suggesting trying to perform the movements with as little effort as possible, providing breaks and asking participants to perform relaxing hand and arm movements between the trials.

Due to organizational constraints, the experiment had to be split between two labs (Berlin and Eugene, see author affiliations of Julius Verrel and Marjorie Woollacott). One of the authors (Marjorie Woollacott) was present during the experiments in both labs, ensuring consistency of experimental procedures. Of the 20 participants, eight were tested in Berlin (six novices, two experts) and twelve in Eugene (four novices, eight experts). Kinematic data were acquired using 3D motion capture systems (Berlin: Vicon MX, Oxford, UK, sampling rate 120 Hz; Eugene: Motion Analysis, PEAK Performance Technologies, Englewood, CO, USA, sampling rate 60 Hz). The data acquired in Berlin were down sampled to 60 Hz during preprocessing.

**FIGURE 1:**
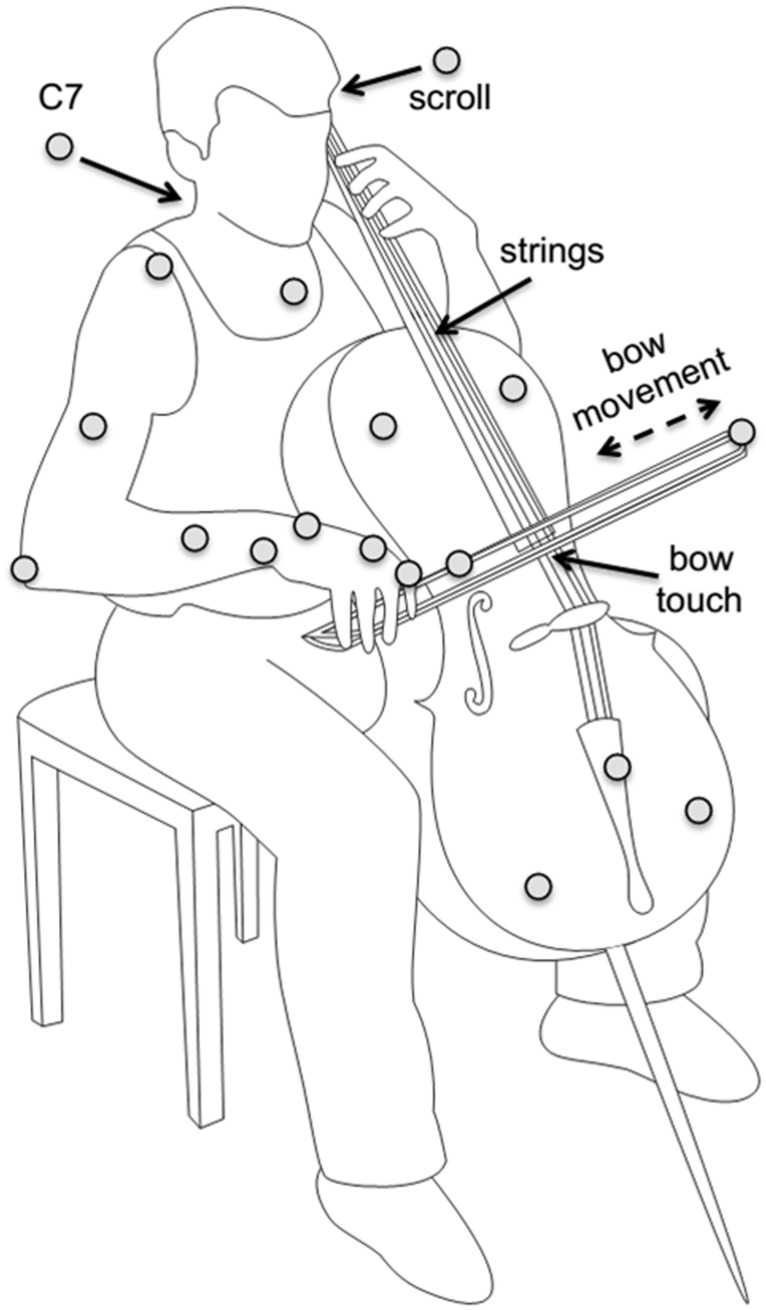
**Illustration of setup, instrument, and task.** Marker positions are indicated as gray circles. Reproduced with permission from Verrel et al. (2013a). Coordination of degrees of freedom and stabilization of task variables in a complex motor skill: expertise-related differences in cello bowing (Verrel et al., 2013a).

Participants wore sleeveless shirts to allow marker placement on the shoulders and upper trunk. Passive reflective markers (diameter 12 mm) were attached directly on the skin of participants on the trunk (sternum, C7), right arm (acromion, lateral epicondyle of the elbow, lower arm, and wrist), and right hand (first metacarpophalangeal joint and first proximal interphalangeal joint). Cello and bow motion were recorded with additional markers on the cello (scroll and tail piece, defining the “string axis”, and on the cello body, defining the lateral axis) and on the bow (on the tip, and about two-thirds of the way between tip and frog); see **Figure 1**.

The task consisted of repeated bowing movements on the open A-string at a metronome-paced tempo of 80 bows per minute (0.75 per bow), that is, 40 down-bows and 40 up-bows per minute. Participants were instructed to try producing a continuous tone, emphasizing smooth and quick transitions between up-bows and down-bows. Twenty bowing cycles (up- and down movements) were acquired per participant.

### DATA PREPROCESSING

Kinematic data were low-pass-filtered with a bidirectional 5th-order Butterworth filter with a cut-off frequency of 20 Hz. Details of the initial data processing have been reported previously (Verrel et al., 2013a). The kinematic data were segmented into bow movements based on bow velocity (Verrel et al., 2013a). Only movements between detected bow reversals with a duration between 0.5 and 1 s (instructed duration: 0.75 s) were scored as bowing movements, and only the first ten up-bow and down-bow movements without missing samples (and the corresponding bow reversals) were analyzed for each participant. Bow reversals were analyzed in time windows of 45 samples (0.75 s) around the time of bow reversal.

From the bow movement data, the following dependent variables were computed: bow movement amplitude, variability of bow angle, variability of bow velocity, and acceleration amplitude. Bow angle variability was quantified by the standard deviation, based on the premise that a constant angle (of around 90°) of the bow relative to the string should be maintained during bow movements (Mantel, 1995). Bow velocity variability was analyzed in two ways: *within-bow variability* was quantified by the average within-bow variance (during the central 80% of a bow movement, i.e., excluding bow reversals); *between-bow variability* was quantified by the average between-bow variance (at each time point of the central 80% of bow movements). Low within-bow variability scores for bow velocity indicate more rectangular velocity profiles, reflecting near-constant bow velocity to ensure a stable tone during bow movements, while more bell-shaped velocity profiles (as found in typical point-to-point movements, e.g., Flash and Hogan, 1985) would result in larger variability scores. Low between-bow variability scores for bow velocity indicate consistent bow movement velocity profiles across bow movements. Both for bow angle and bow velocity variability, higher variability is assumed to reflect poorer performance, as confirmed in a previous study (Verrel et al., 2013a). More details on the computation and rationale for these measures can be found elsewhere (Verrel et al., 2013a,b).

Based on anthropometric measures (elbow, wrist, and hand thickness), approximate joint center positions were computed and added as “virtual markers” for further analysis. Local coordinate systems (LCS) were defined for scapula, upper arm, lower arm, hand, and bow based on a previously described method (Verrel et al., 2012). Briefly, the LCS was defined by applying the Gram-Schmidt process to two well-defined vectors relative to the body segment. For the scapula, the vectors were defined by sternum and shoulder joint center (primary axis) as well as the the cross product of the global vertical with the primary axis. For the lower arm, the LCS primary axis was defined by the elbow joint and the wrist joint, and the secondary axis as the cross-product between the primary axis and the line between the two wrist markers. For the upper arm, the primary axis of the LCS was defined by the shoulder and elbow joints and the secondary axis by the cross-product with the primary axis of the lower arm (Rab et al., 2002). The latter is only possible as long as the elbow does not hyperextend, which indeed did not occur in the present task. For the hand segment, the primary axis was defined by wrist and finger (first metacarpophalangeal) joint and the secondary axis was defined by the cross-product of the primary axis with the line between the two wrist markers. For the bow, the Gram–Schmidt process was applied to the primary axis defined by the two bow markers as well as the vector from the proximal bow marker to the distal finger marker.

Subsequently, relative configurations of neighboring segments along the kinematic chain were expressed as rigid body motions (translation and rotation). This joint-level representation allows reconstructing the original data by reverting the above computation, from joint configurations to marker positions. Importantly, this is also possible for surrogate data, in which movement at a particular joint is changed (“frozen”, i.e., set to a constant value) relative to the originally measured movement.

We assessed the contribution of motion at individual joints on bowing performance by artificially “freezing” (setting to constant) the configuration of that joint and comparing the resulting surrogate data to the original data in terms of the performance measures. For performance measures related to the entire bow movement (amplitude, bow angle variability, bow velocity variability), the joint configuration was set constant to the value at the midpoint of the first analyzed bow movement in a trial. For performance measures related to bow reversal (acceleration amplitude), the joint configurations was set constant to the configuration at the time of the first analyzed bow reversal.

This analysis was performed for shoulder, elbow, and wrist joint. The effect of freezing a joint on performance was quantified by the log10 of the ratio between surrogate and original data for the different performance variables. On this scale, positive values indicate relative increases in the performance variable in surrogate compared to original data, and negative values indicate decreases.

### STATISTICAL ANALYSIS

Statistical analyses were performed in R (R Development Core Team, 2008; Lawrence, 2011) with a significance threshold of 0.05. To assess the effect of freezing individual joints, relative scores (log10 of the ratio between surrogate and original data) were tested for being different from 0 using one-sample *t*-tests. This was done separately for each joint and bow direction, adjusting *p*-values for multiple comparisons (Holm, 1979). Group differences in freezing effects were assessed separately for each joint and bow direction, again adjusting for multiple comparisons. In order to control for the confound between Group and Lab, this was done using a between-subject ANOVA (Type II) with factors Group and Lab. Only the effects of Group are reported, as preliminary analyses showed that the effect of Lab did not reach significance in any case and significant main effects of Group (reported below) were not undermined by Group x Lab interaction effects.

## RESULTS

### SAMPLE DATA AND ILLUSTRATION OF FREEZING ANALYSIS

**FIGURE 2:**
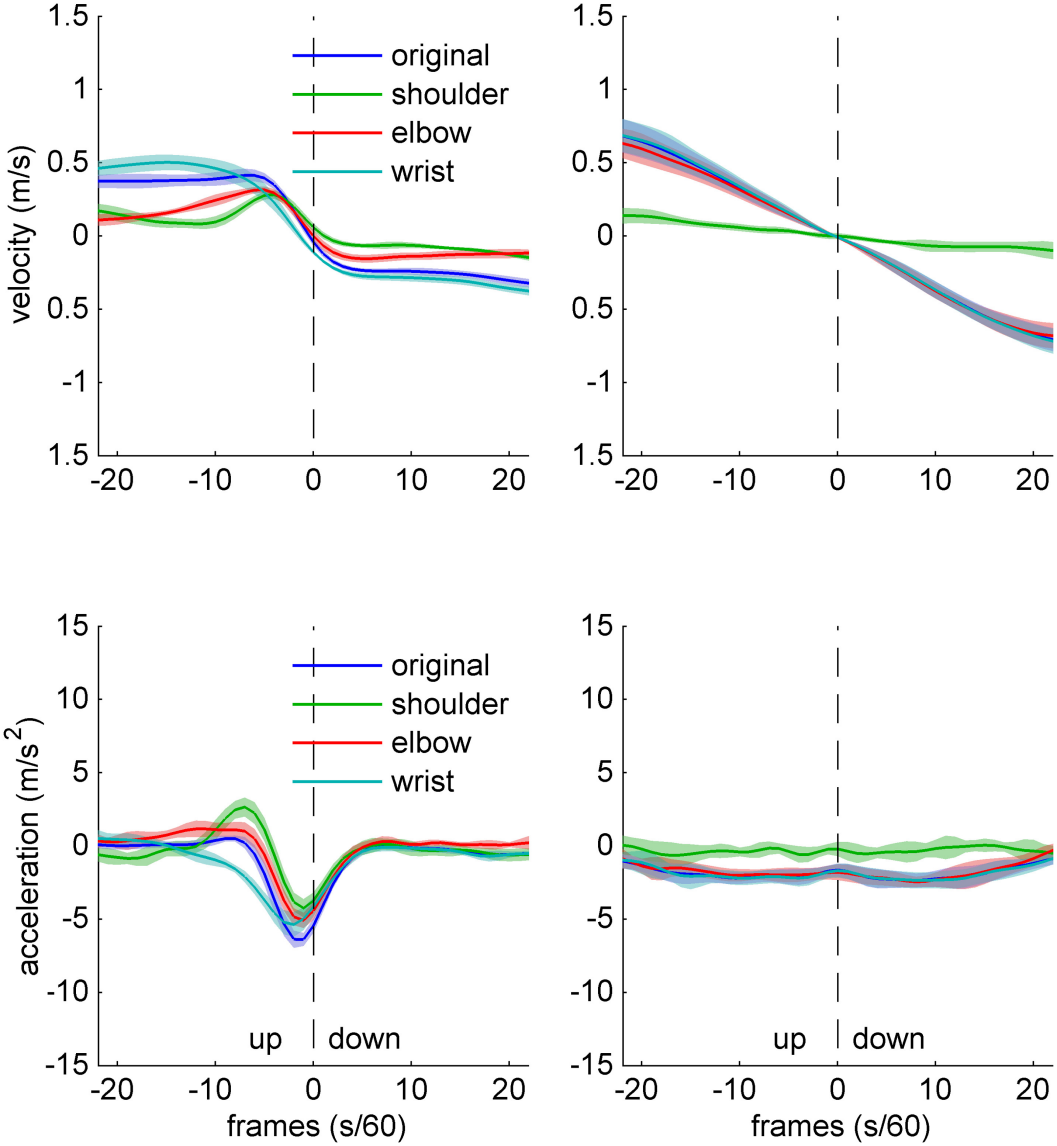
**Sample data of bow kinematics (velocity and acceleration) and joint angle movement for an Expert (left) and a Novice (right) cello player, during an up–down bow reversal.** The different traces indicate original data (blue), as well as surrogate data created by artificially removing movement at the shoulder (green), elbow (red), and wrist (turquoise). Shaded areas indicate SD across movements.

The freezing analysis is illustrated in **Figure 2**, which shows bow velocity and acceleration data for one expert (left panel) and one novice (right panel) participant, during an up–down bow reversal. The individual traces show original data, as well as surrogate data which were obtained by artificially removing movement at (“freezing”) individual joints. The sample data suggest that, freezing any of the three joints has marked effects on velocity and acceleration profiles in the expert, while in the novice, only freezing the shoulder joint does seem to have any effect.

### EFFECT OF “FREEZING” ON BOW KINEMATICS DURING BOW MOVEMENTS

**FIGURE 3:**
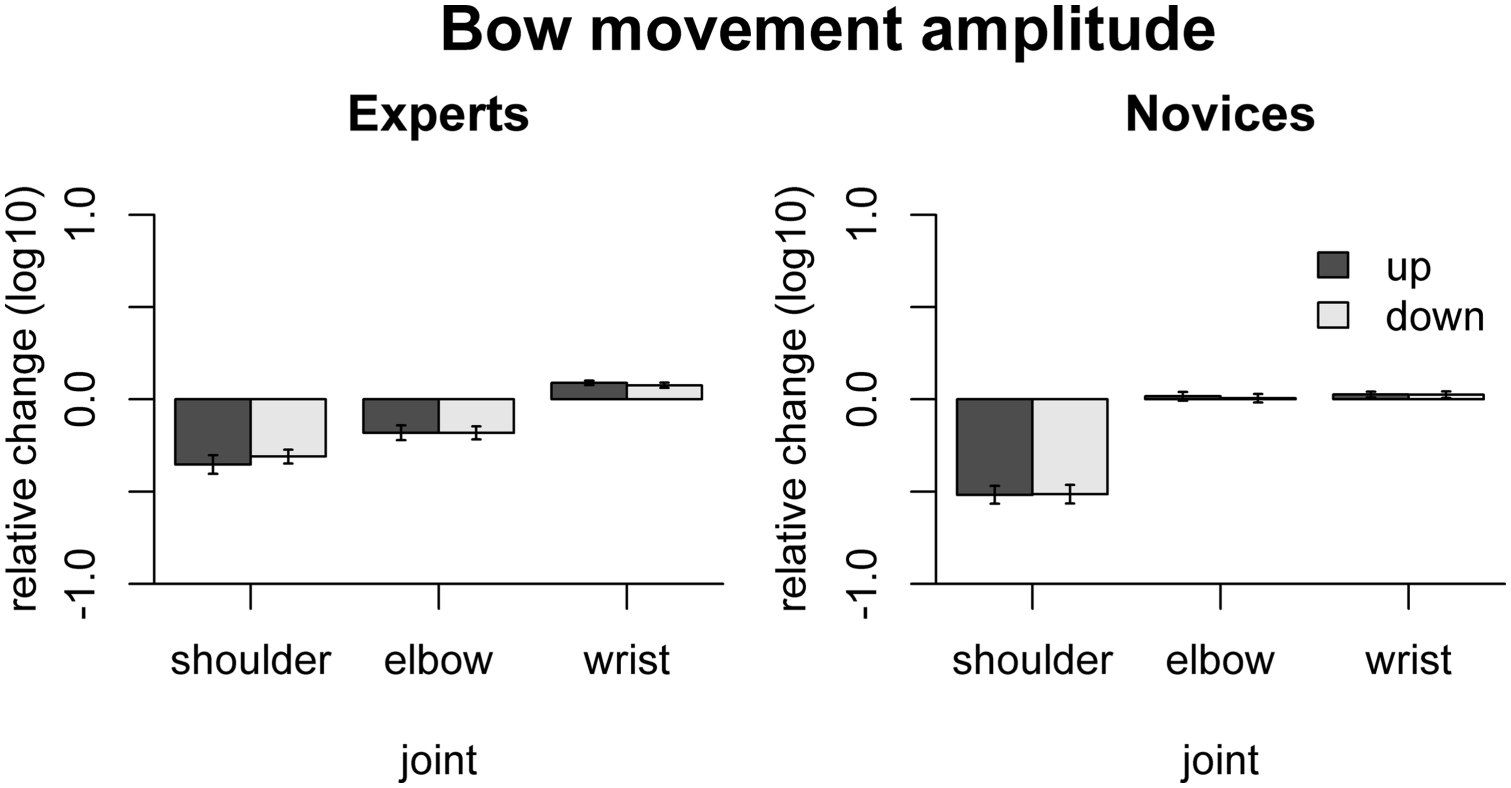
**Results of the freezing analysis for bow movement amplitude.** The relative change is computed as the log10-transformed ratio between surrogate and original data. Thus, positive values indicate that freezing a particular joint leads to an increase in movement amplitude. Error bars indicate SE across participants.

Group results for the freezing analysis with respect to bow movement amplitude are shown in **Figure 3**. In experts, freezing led to significant amplitude reduction for the shoulder [up-bows: *t*(9) = -7.02, *p*_adj_ < 0.001, down-bows: *t*(9) = -8.33, *p*_adj_ < 0.001] and the elbow joint [up-bows: *t*(9) = -4.51, *p*_adj_ = 0.0015, down-bows: *t*(9) = -5.21, *p*_adj_ = 0.0014], while freezing the wrist induced an amplitude increase [up: *t*(9) = 7.26, *p*_adj_ < 0.001, down: *t*(9) = 5.34, *p*_adj_ = 0.0014]. In the novices, only freezing the shoulder induced any change, namely an amplitude reduction [up: *t*(9) = -10.62, *p*_adj_ < 0.001, down: *t*(9) = -10.1, *p*_adj_ < 0.001]. Comparison between experts and novices showed that the effects of freezing the shoulder were more pronounced in novices than in experts for [up: *F*(1,16) = 7.64, *p*_adj_ = 0.041, down: *F*(1,16) = 11.6, *p*_adj_ = 0.014], while freezing effects were more pronounced in experts for the elbow [up: *F*(1,16) = 14.2, *p*_adj_ = 0.008; down: *F*(1,16) = 15.6, *p*_adj_ = 0.007].

Thus, both shoulder and elbow contribute to bow transport in experts while only the shoulder contributes to bow transport in novices. The fact that freezing the wrist in experts leads to an increase in movement amplitude shows that the wrist does not positively contribute to bow transport, but actually performs a counter-movement, which may be related to control of other bow parameters.

**FIGURE 4:**
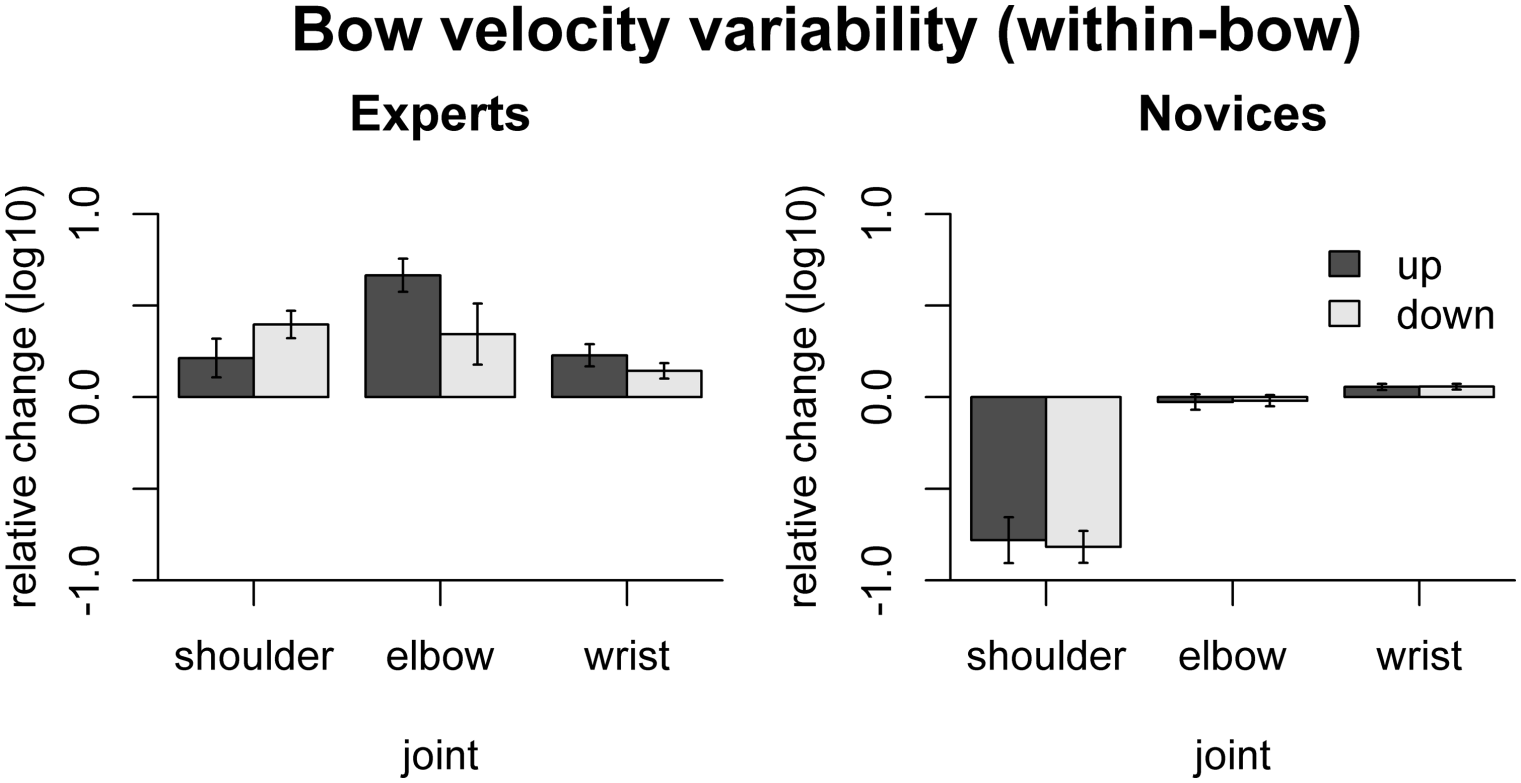
**Results of the freezing analysis for within-bow variability of bow velocity (central 80% of the bow movement).** Error bars indicate SE across participants.

The effect of freezing on *within-bow variability* of bow velocity is shown in **Figure 4**. In experts, variability was increased by freezing the shoulder [only down: *t*(9) = 5.30, *p*_adj_ = 0.003], elbow [only up: *t*(9) = 7.36 *p*_adj_ = 0.0026], or wrist [up: *t*(9) = 3.80 *p*_adj_ = 0.017, down: *t*(9) = 3.41, *p*_adj_ = 0.023]. In novices, in contrast, bow velocity variability was *reduced* by freezing the shoulder joint [up: *t*(9) = -6.23, *p*_adj_ < 0.001, down: *t*(9) = -9.37, *p*_adj_ < 0.001] and *increased* by freezing the wrist joints [up: *t*(9) = 3.34, *p*_adj_ = 0.026, down: *t*(9) = 3.68, *p*_adj_ = 0.02]. Comparison between the groups showed that the (variance-increasing) effects of freezing joints were more pronounced in experts than in novices for shoulder [up: *F*(1,16) = 25.8, *p*_adj_ < 0.001, down: *F*(1,16) = 92.1, *p*_adj_ < 0.001] and elbow [only up: *F*(1,16) = 44.7, *p*_adj_ < 0.001].

**FIGURE 5:**
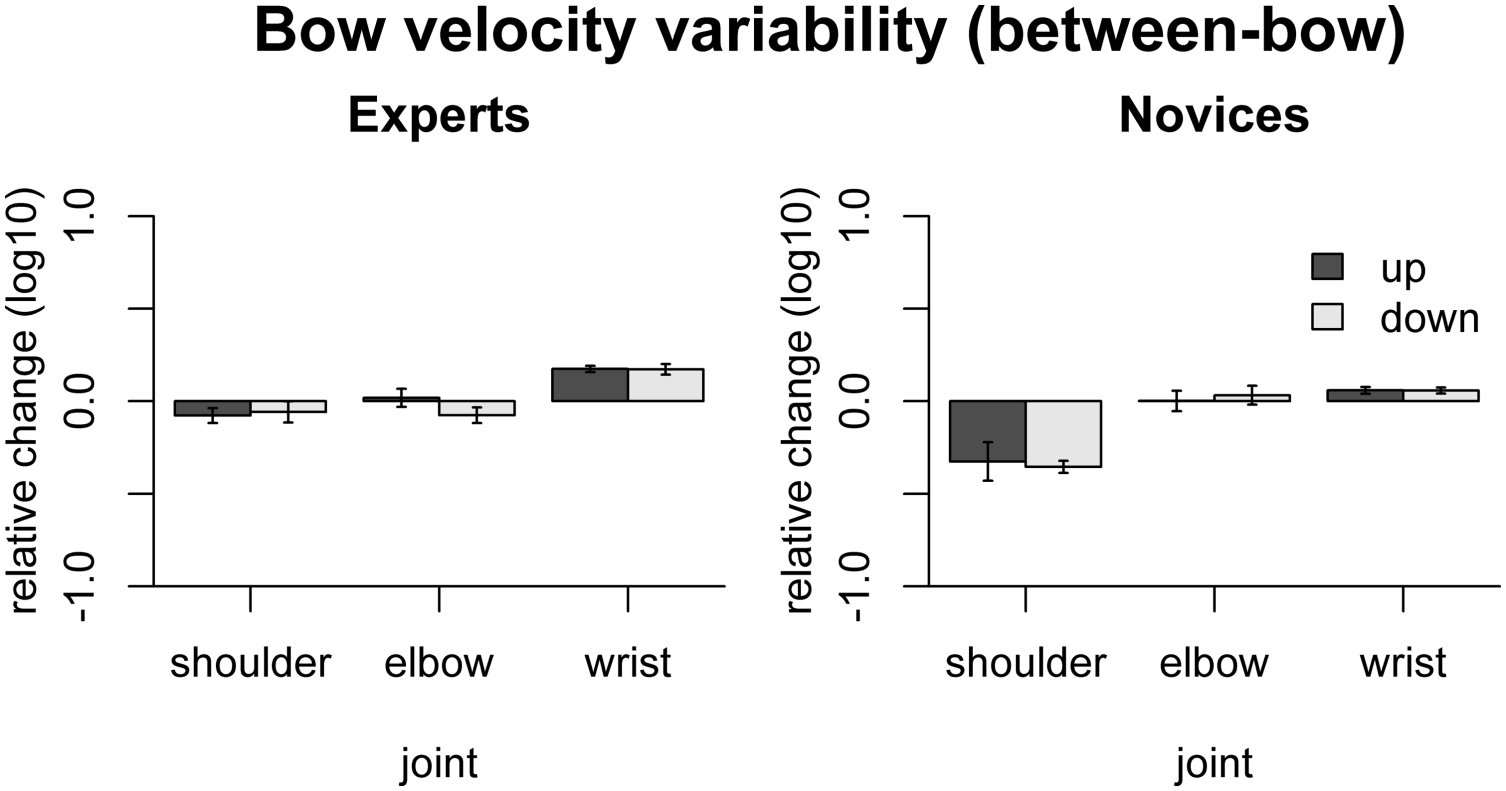
**Results of the freezing analysis for between-bow variability of bow velocity (central 80% of the bow movement).** Error bars indicate SE across participants.

The effect of freezing on *between-bow variability* of bow velocity is shown in **Figure 5**. In experts, variability was increased by freezing the wrist [up: *t*(9) = 10.29 *p*_adj_ < 0.001, down: *t*(9) = 6.08, *p*_adj_ < 0.001]. In novices, in contrast, bow velocity variability was *reduced* by freezing the shoulder joint [up: *t*(9) = -3.11, *p*_adj_ = 0.045, down: *t*(9)= -10.7, *p*_adj_ < 0.001] and *increased* by freezing the wrist joints [up: *t*(9) = 3.18, *p*_adj_ = 0.045, down: *t*(9) = 3.44, *p*_adj_ = 0.037]. Comparison between the groups showed that the variance-decreasing effect of freezing joints was more pronounced in novices than in experts for the shoulder [only down: *F*(1,16) = 19.55, *p*_adj_ = 0.002], and the variance-increasing effect was more pronounced in experts than in novices for the wrist [up: *F*(1,16) = 24.2, *p*_adj_ < 0.001, down: *F*(1,16) = 13.0, *p*_adj_ = 0.010].

These results indicate that in experts, shoulder, elbow, and wrist are coordinated to achieve a near-constant, rectangular velocity profile (minimizing *within-bow variability).* Moreover, in experts the wrist motion appears to be crucial for maintaining a consistent bowing pattern across bows (minimizing *between-bow variability)*. Most of these effects are less pronounced, absent, or even go in the opposite direction in the novices.

**FIGURE 6:**
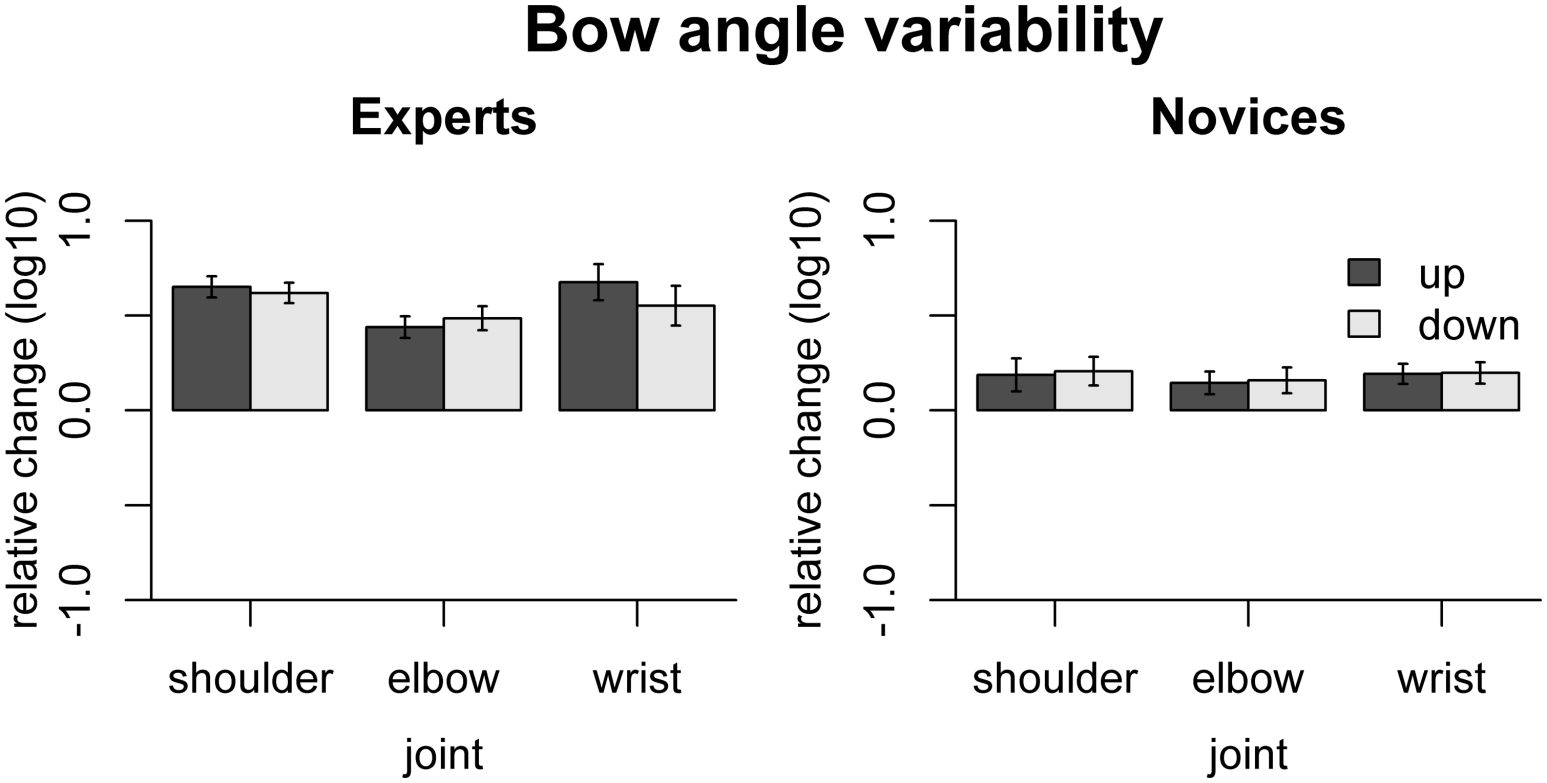
**Results of the freezing analysis for bow angle variability.** Error bars indicate SE across participants.

The effect of freezing on variability of bow angle is shown in **Figure 6**. In experts, freezing any of the joints increased bow angle variability, shoulder [up: *t*(9) = 11.69, *p*_adj_ < 0.001, down: *t*(9) = 11.56, *p*_adj_ < 0.001], elbow [up: *t*(9) = 7.66, *p*_adj_ < 0.001, down: *t*(9) = 7.68, *p*_adj_ < 0.001], and wrist [up: t(9) = 7.12, *p*_adj_ < 0.001, down: *t*(9) = 5.27, *p*_adj_ < 0.001]. In contrast, in novices, only freezing the wrist joints led to significantly increased bow angle variability [up: *t*(9) = 3.63, *p*_adj_ = 0.032, down: *t*(9) = 3.49, *p*_adj_ = 0.034]. The effects of freezing were more pronounced in experts than in novices for each of the joints: shoulder [up: *F*(1,16) = 16.25, *p*_adj_ = 0.0058, down: *F*(1,16) = 16.06, *p*_adj_ = 0.0058], elbow [up: *F*(1,16) = 15.13, *p*_adj_ = 0.0058, down: *F*(1,16) = 13.37, *p*_adj_ = 0.0058], and wrist [up: *F*(1,16) = 13.86, *p*_adj_ = 0.0058, down: *F*(1,16) = 4.99, *p*_adj_ = 0.040].

The fact that freezing of any joint led to a significant increase in bow angle variability in experts indicates that all the joints are coordinated in a way stabilizing bow angle variability. This was not the case in novices, or to a much lesser extent, as only freezing the wrist joint increased bow angle variability.

### EFFECT OF “FREEZING” ON ACCELERATION AT BOW REVERSALS

**FIGURE 7:**
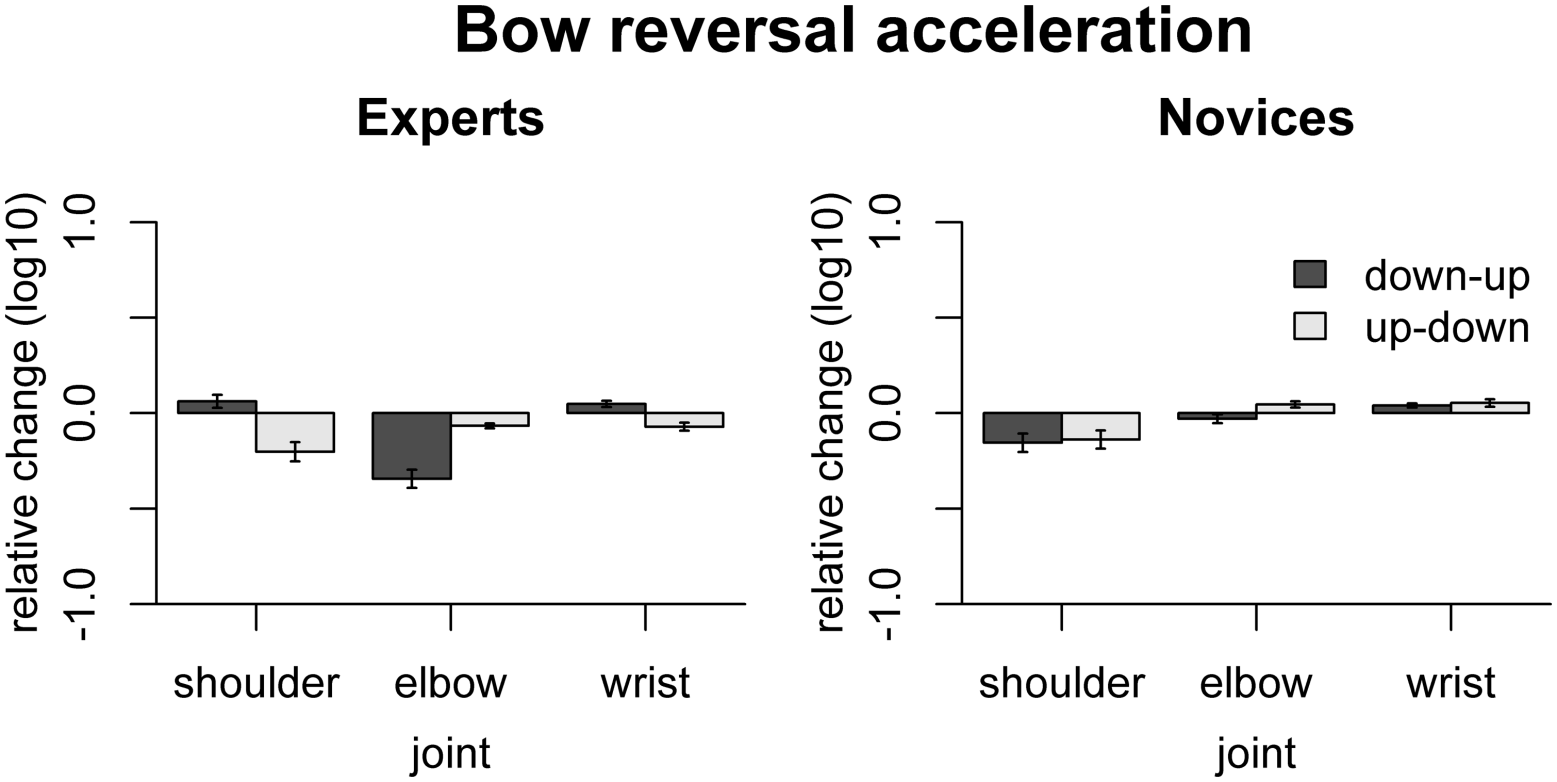
**Results of the freezing analysis for bow acceleration amplitude.** Error bars indicate SE across participants.

The effect of freezing on variability of acceleration at bow reversals is shown in **Figure 7**. In experts, freezing the shoulder reduced acceleration for up–down reversals [*t*(9) = -4.0, *p*_adj_ = 0.012], freezing the elbow reduced acceleration both for up–down and down–up reversals [up–down: *t*(9) = -5.25, *p*_adj_ = 0.0026, down–up: *t*(9) = -7.23, *p*_adj_ < 0.001], and freezing the wrist reduced acceleration for up–down reversals [*t*(9) = -3.41, *p* = 0.023] but increased acceleration for down–up reversals [*t*(9) = 3.01, *p* = 0.029]. In novices, only freezing the wrist had a significant effect, namely to increase acceleration in down–up reversals [*t*(9) = 3.54, *p* = 0.037]. The effects of freezing differed between the groups for freezing the shoulder for down–up reversals [*F*(1,16) = 21.6, *p*_adj_ = 0.001], for freezing the elbow for both bow change directions [up–down: *F*(1,16) = 22.66, *p*_adj_ = 0.0011, down–up: *F*(1,16) = 23.77, *p*_adj_ = 0.001], and freezing the wrist for up–down reversals [*F*(1,16) = 22.62, *p*_adj_ = 0.001].

The observed effects of freezing on bow acceleration indicate that, in experts, the shoulder and wrist mostly contribute to quick up–down reversals, while the elbow contributes to quick reversals in both directions. In both groups, freezing the wrist increased acceleration in down–up reversals, suggesting that actual movement of the wrist does not contribute to achieving high acceleration amplitudes for down–up reversals.

## DISCUSSION

We used a novel “freezing” analysis to investigate how coordinated movement at different joints of the right arm during cello playing contributes to bow transport, stabilization of bow parameters during bow movements, and quick bow reversals. This analysis showed that several or all joints contributed to performance in expert cellists. Most corresponding effects were less pronounced or absent in novices, in particular for the more distal joints. The results are discussed in more detail below.

### SKILLED PERFORMANCE DEPENDS ON DISTRIBUTED USE AND COORDINATION OF DEGREES OF FREEDOM

Applying the freezing analysis in a similar way to that previously suggested by Park et al. (2005), we showed that both shoulder and elbow contribute to bow transport in experts, as eliminating motion at these joints led to a reduction of movement amplitude in surrogate compared to original data. In contrast, only the shoulder contributed to bow transport in novices. Thus even this basic aspect of bowing performance, which is very similar to point-to-point movements encountered in everyday life, shows a marked difference between experts and novices with regard to the contribution of individual joints. Interestingly, the wrist did not positively contribute to bow transport in the experts, but actually showed a counter-movement (indicated by the amplitude decrease in the frozen data). It is plausible that movement at the wrist, with a relatively small lever with respect to the bow, may not serve to maximize bow movement amplitude but to control other aspects of performance in expert cello players.

This view is supported by the analysis concerning variability of bow velocity and bow angle. In the expert participants, freezing any of the joints could lead to significant increases in performance variability, indicating that they were originally coordinated in a way minimizing this variability. Within-bow variability of bow velocity was most strongly increased by freezing the shoulder or elbow, which is consistent with the dominant role of these joints for bow transport. Between-bow variability of bow velocity was most strongly affected by freezing the wrist joint. In contrast, bow angle variability was affected by freezing any of the three joints, indicating that coordinated motion across all three joints was essential for stabilizing bow angle. In the novices, these effects were less pronounced, absent, or even went in the opposite direction, indicating that coordination among joints was more important in experts than in novices for stabilizing bow velocity and bow angle.

These findings are consistent with the leading joint hypothesis (Dounskaia, 2005), according to which the dynamic foundation of motion in multi-degree-of-freedom movements is primarily generated by a “leading joint” (e.g., the shoulder or elbow for bow transport in the present task) while muscles at other joints exploit and finely regulate interaction torques generated by the leading joint in order to control other task parameters (e.g., bowing angle). Support for this hypothesis has been found across multiple motor behaviors, including ball throwing (Hirashima et al., 2003), and generating piano key strokes (Furuya and Kinoshita, 2008).

The freezing analysis regarding bow acceleration showed that, in experts, the shoulder and wrist mostly contribute to quick up–down reversals, while the elbow contributes to the speed of both down–up and up–down reversals. One may speculate that the difference of the contribution of the wrist between up–down and down–up reversals is due to biomechanical differences between the two movement directions. Differentiated use of the wrist, which has been characterized as part of a whip-like movement (Mantel, 1995; Verrel et al., 2013b) may be particularly important when the reversal is initiated from a more proximal joint (as the shoulder in up–down reversals), at which higher angular accelerations would induce larger interaction torques with the rest of the body. Unfortunately, this question cannot be directly addressed by the present, purely kinematic analysis. No corresponding effects were found in the novices, and the group comparison emphasizes the importance of elbow motion in down-up reversals and wrist motion in up–down reversals for expert performance.

Our present results may appear inconsistent with a previous study reporting that learning to play the violin was associated with freezing rather than freeing of degrees of freedom (Konczak et al., 2009). Focusing on shoulder and elbow motion, Konczak et al. (2009) observed a reduction in shoulder range of motion and no systematic change in elbow range of motion with increasing expertise. This partial result is actually consistent with our current and previous findings with cello players (Verrel et al., 2013a,b), showing mostly proximal (shoulder) movement in novices and a more distributed coordination pattern in experts, involving in particular more distal joints (elbow and wrist). Unfortunately, our results, which provide clear evidence for expertise-related freeing of degrees of freedom, cannot be compared to Konczak et al. (2009) results due to methodological shortcomings of their study. First, due to technical problems, Konczak et al. (2009) were unable to analyze wrist motion, which is non-negligible in stringed instrument bowing, both according to our present and previous results on cello playing (Verrel et al., 2013a,b), kinematic analysis of violin bowing (Turner-Stokes and Reid, 1999), and cello and violin pedagogy (Mantel, 1995; Starr, 1996). Moreover, Konczak et al. (2009) compare joint movement amplitudes (of shoulder and elbow) across participants of a wide age range (4–47 years), without controlling for age (and hence body size) and bow movement amplitude, which both are likely to be confounded with expertise.

Summing up, we found evidence for enhanced use and coordination, in particular of distal joints (wrist and elbow), with respect to the performance variables under consideration, in expert compared to novice cello players. Our findings support the theory of skill acquisition proposed by Nikolai Bernstein (Bernstein, 1967, 1996), suggesting that acquiring a new complex skill is characterized by initial freezing of degrees of freedom, followed by a gradual release of degrees of freedom in the service of more accurate and efficient performance. Our findings more specifically show the importance of integrating distal degrees of freedom, as previously empirically found for the tasks of hand writing (Newell and Van Emmerik, 1989) and racquet ball performance (Southard and Higgins, 1987), and which has also been described in the literature on cello technique (Mantel, 1995). The change from more proximal to a more distributed use of degrees of freedom is functionally plausible in cello bowing. The main requirement for producing a tone on the cello is bow transport, which requires a large-amplitude movement most easily achieved by a proximal joint (the shoulder, as observed in the novices). However, generating the bow movement solely from the shoulder would not allow stabilizing additional task parameters (in particular bowing angle). Moreover, it has been argued that a distributed and temporally differentiated coordination pattern across degrees of freedom may be crucial for movement efficiency, in particular when high velocity or acceleration patterns need to be achieved (Southard and Higgins, 1987; Putnam, 1993; Hirashima et al., 2003; Furuya and Kinoshita, 2007; Verrel et al., 2013b).

As a limitation of the present study (and any studies using purely movement-based analyses of music performance), we acknowledge that the ultimate goal of cello bowing is production of muscial expression, rather than a particular movement trajectory *per se*. Thus, the performance measures used in the present study (e.g, amplitude of bow acceleration amplitude, variability fo bow velocity and bow angle) can only be considered approximations to actual music performance, which are nevertheless based on the cello pedagogy literature (Mantel, 1995).

### METHODOLOGICAL CONSIDERATIONS

The freezing analysis used in the present paper was devised to address the methodological problem of relating continuous motion at different degrees of freedom (joint angles) to motion at an end effector (the bow). To this end, we created surrogate data, in which motion at individual joints is artificially removed, and compared the resulting performance (in terms of task-specific, quantitative performance measures) to the original data. Applied to assess the contribution of different joints to bow transport, our approach is similar to previous studies (Park et al., 2005, 2008). However, the application to variability and acceleration measures presents a methodological innovation of the present paper.

Approaches relating variability at joints (or other “elemental variables”) to variability at end effectors have been proposed (Scholz and Schöner, 1999; Müller and Sternad, 2003; Verrel, 2011), but these are only applicable for discrete time points (e.g., to assess trial-to-trial variability) or quasi-static tasks (e.g., fluctuations around a constant average posture). Meaningful generalizations of these approaches to coordination between degrees of freedom during a movement are not evident.

The presently used analysis has the intuitive appeal of mimicking the proposed freezing and freeing of degrees of freedom during skill acquisition (Bernstein, 1967). Our approach simulates what performance would be like, if a particular joint was eliminated or “frozen” during a movement, with all the other joints unchanged. Comparing this simulated movement to the originally observed movement allow the direct testing of the hypothesis that use and coordination of this joint with the other joints matters for task performance. The freezing analysis thereby operationalizes the problem of motor equivalence, that is, the exploitation of abundant degrees of freedom to stabilize performance in the presence of intended movement and variability/perturbation within the system. Successful cello bowing requires bodily movement (for bow transport) while concurrently stabilizing certain task variables (e.g., bow angle), and the present results confirm that – especially experts – do make use of abundant degrees of freedom to ensure successful performance.

However, this approach also has limitations. In particular it is not obvious that the resulting surrogate data are realistic in the sense that they are similar to empirical data resulting from actual freezing of joints in early stages of skill acquisition. For instance, due to biarticular muscles (spanning more than one joint), co-contraction of muscles around a joint does not necessarily result in a simple elimination of motion at a particular joint. Moreover, the present approach is purely kinematic and does not take into account joint torques (nor interaction torques between body segments), which actually generate the physical movement. Thus, the freezing approach does not directly assess the contribution of torques generated at one or multiple joints to task performance. However, by taking into account the geometric relationship between degrees of freedom and task variables, it goes beyond commonly used kinematic approaches. For instance, previous results show that angular motion is strongly reduced in novice compared to expert cello players at distal arm joints (Verrel et al., 2013a). The present analysis allows testing how eliminating motion at particular degrees of freedom (irrespective of the underlying joint dynamics) affects kinematic performance measures.

Artificially freezing motion at individual joints may result in physiologically unrealistic data, thereby potentially introducing artifacts. The present approach shares this limitation with any analysis method based on generation of surrogate data, as it is not known which are the independent “elemental variables” (e.g., spatial positions, joint angles, muscle activations, joint torques) in terms of which the central nervous system controls movements (Feldman and Levin, 1995; Gribble et al., 1998; Pandy, 2001; d’ Avella et al., 2003). In the present approach, unrealistic surrogate data may be expected at bow reversals (amplitude of acceleration), during which mechanical interactions between body segments are likely to be most prominent due to the quick reversal (in experts). Thus, the results for the amplitude of acceleration should be interpreted more cautiously than for the other performance measures, defined during the bow movement.

Depending on the performance measures used, the freezing analysis addresses the *contribution* of individual joints to change in a task variable (e.g., bow movement amplitude) or the *coordination* of joints with for stabilizing a task variable (e.g., bow angle). Artificially eliminating motion at individual joints has the dual effect of reducing the overall motion in the system and removing coordination of that joint with the other joints. For performance measures concerning movement (or acceleration) amplitude, reductions due to the freezing manipulation can be both due to reduced motion *or* reduced coordination. Hence, freezing effects observed for a particular joint with respect to bow movement or acceleration *amplitude* do not necessarily reflect *coordination* of that joint with the other joints, but may be mostly due to individual *contribution* of that joint to performance.

The situation is different for variability-based performance measures. The relationship between joint angles and performance variables can be described by a geometric forward model. In the absence of coordination (the null-hypothesis), movement at one joint does not significantly influence the effect of another joint on the performance variable. Thus, the *a priori effect* of eliminating motion at one joint would be (based on the application of the forward model) a reduction in the amount of motion at the performance level (e.g., bow angle), and hence a reduction in performance variability. This means that, in contrast to amplitude-based performance measures, an *increase* in performance variability can only be due to elimination of coordination between joints. Thus, if freezing a particular joint (e.g., the wrist) leads to an increase in variability in a task variable (e.g., bow angle), this indicates that this joint was originally *coordinated* with the other joints in a way stabilizing the task variable under consideration. The effects of eliminating individual variation (movement at particular joints) versus eliminating covariation (coordination across joints) cannot entirely be separated in the present approach. However, the expected effects are opposite for variability- based performance measures, as discussed above. Thus, the freezing analysis assesses between-joint coordination with respect to performance variability conservatively.

## CONCLUSIONS

We used a novel “freezing” analysis to investigate how coordinated movement at different joints contributes to bow transport, stabilization of bow parameters during bow movements, and quick bow reversals. This analysis showed that several or all joints contributed to performance in expert cellists. In contrast, corresponding effects were less pronounced or absent in novices. Our results demonstrate the importance of distributed use and coordination of the joints of the right arm for experts’ bowing performance and support theories of motor control and learning which propose that exploitation of biomechanical degrees of freedom, in particular of distal joints, is crucial for skilled motor performance.

## Conflict of Interest Statement

The authors declare that the research was conducted in the absence of any commercial or financial relationships that could be construed as a potential conflict of interest.
